# Spontaneous reporting of adverse drug reaction among health professionals in Ghana

**DOI:** 10.1186/s13690-021-00783-1

**Published:** 2022-01-20

**Authors:** Morrison Asiamah, Kwadwo Owusu Akuffo, Pricillia Nortey, Nina Donkor, Anthony Danso-Appiah

**Affiliations:** 1 Department of Epidemiology and Disease Control, School of Public Health, College of Health Sciences, University of Ghana, Legon, Ghana; 2 Department of Optometry and Visual Science, College of Science, Kwame Nkrumah University of Science and Technology, Kumasi, Ghana; 3 Department of Pharmacy Practice, College of Health Sciences, Kwame Nkrumah University of Science and Technology, Kumasi, Ghana; 4 University of Ghana Centre for Evidence Synthesis and Policy, School of Public Health, University of Ghana, Legon, Accra, Ghana

**Keywords:** Adverse drug reaction, Reporting, Health professionals, Ghana

## Abstract

**Background:**

Spontaneous reporting of adverse drug reactions (ADR) is an effective means of ensuring postmarketing surveillance of drugs, and health professionals play a cardinal role through voluntary reporting of ADR. However, the pharmacovigilance system in Ghana is plagued with under-reporting issues, which is of public health concern.

**Method:**

A questionnaire-based cross-sectional study involving 268 health professionals at Kpone-Katamanso District was carried out. Data on spontaneous reporting of ADR, demographics of participants, knowledge, and attitudes of professionals towards reporting and factors that may influence ADR reporting were collected. Logistic regression models were used to examine the association of the independent variables with spontaneous reporting of ADR.

**Result:**

Overall, 77.6% (208) of the 268 respondents had observed ADR; however, only 17.3% of the respondents had ever reported an ADR to the Ghana FDA. Health professionals who had average knowledge on spontaneous reporting of ADR were 51.9%, while 30.3% had good knowledge of spontaneous reporting of ADR. After adjustment on potential confounding variables (Knowledge, Feedback from FDA, Uncertainty about cause of ADR, Severity of ADR), Age (AOR = 2.26, 95%CI = 1.25–4.10), Fear of Legal Consequences (AOR = 0.15, 95%CI = 0.41–0.51), Time Constraint (AOR = 0.3, 95%CI = 0.10–0.91), Pharmacovigilance training (AOR = 18.78, 95%CI = 5.46–64.59) and Unavailability of Reporting form (AOR = 0.28, 95%CI = 0.09–0.88) were found to be significantly associated spontaneous reporting of ADR.

**Conclusion:**

The proportion of health professionals in the Kpone- Katamanso District who spontaneously reported observed ADR was low though they had average knowledge about ADR reporting. This underscores the need for a policy to be implemented that makes spontaneous reporting of adverse drug reaction mandatory for health professionals.

## Background

Drugs play a significant role in disease management. However, when administered within their therapeutic dosing schedule, drugs can lead to unwanted effects, which may pose threats to the life of patients [1]. These unwanted effects are referred to as adverse drug reactions (ADR) - undesirable effects that may occur as part of a drug’s pharmacological action [2]. The economic and social impact of ADR is huge and cannot be overlooked [3]. ADR has been projected to be one of the leading causes of morbidity and mortality across all age groups and hospital admissions [4, 5].

In order to reduce the incidence of ADR, the safety of drugs are ascertained through various phases of clinical trials prior to their approval on the market [6]. However, the safety reports from these clinical trials are collated from strictly controlled conditions which may be subject to bias due to strict inclusion and exclusion criteria and limited time. For example, the effects of these drugs are often not studied on some members of the population such as children under 5 years, pregnant women and very old people; thus, yielding outcomes that may not be perfectly generalizable [7].

Spontaneous reporting of ADR is the cornerstone of pharmacovigilance that helps address safety concerns after drug administration [8]. It provides information from real-life clinical practice as opposed to that of clinical trials where some subjects are excluded and the safety of the drug is studied under a limited time [3, 9]. Spontaneous reporting is an inexpensive, flexible and very effective method of collecting information whereby health professionals voluntarily submit case reports of ADR; pharmaceutical companies or consumers to the national pharmacovigilance centres for evaluation in order to mitigate the impact of ADR on society [9].

**Fig. 1:**
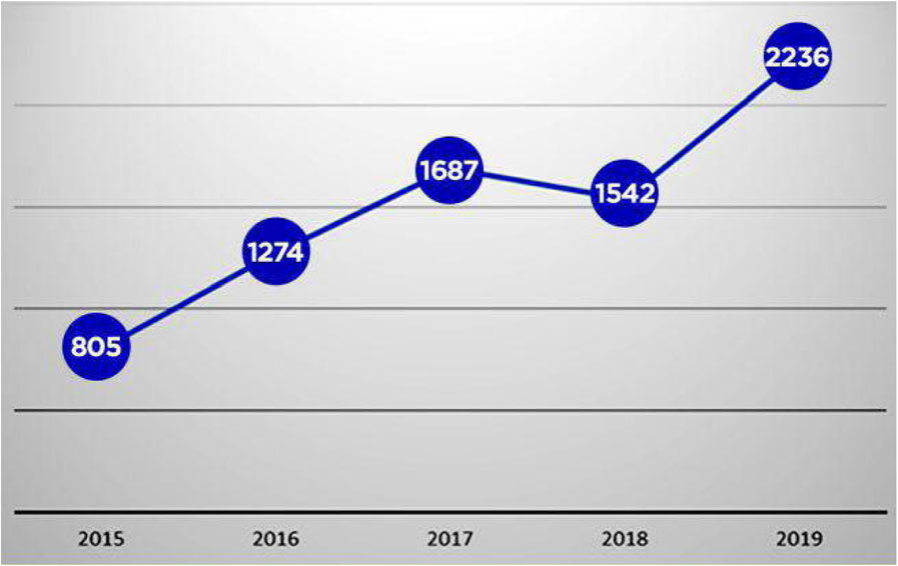
Trend in adverse event reporting in Ghana from 2015 to 2019

For a pharmacovigilance system to effectively monitor ADR of both existing and newly introduced drugs, the World Health Organisation (WHO) recommends that the spontaneous reporting of adverse effects should be more than 200 reported cases per 1,000,000 population [10]. In 2019, the National Pharmacovigilance Center of Ghana recorded 2236 reported cases of adverse drug cases representing about 75 reported cases per 1,000,000 population of Ghanaians [10]. Though an improvement in reported cases from previous years, the reporting rates have constantly been below the WHO standard (Fig. 1). Underreporting of ADR is a big challenge in pharmacovigilance, especially in countries that utilize the spontaneous or voluntary ADR reporting system such as Ghana [11, 12].

Per WHO recommendations, Kpone-Katamanso district, with a huge population and several health facilities, should report a minimum of 22 cases of ADR a year. However, the number of ADR reported, and the factors influencing reporting are largely unknown. Several studies have identified attitudes such as complacency or doubt about the causal relationship of the event with the drug, procrastination and indifference to report, and apprehension about the legal consequences of reporting ADR as attitudes negatively affecting spontaneous reporting [13]. An assurance of confidentiality of the reporter’s identity, legal protection and incentives for reporting have been identified to promote spontaneous reporting [14]. Additionally, indifference, diffidence, complacency, ignorance, lack of monetary incentives, and personal desire to publish cases have been suggested as predictors of low ADR reporting, to the extent that in some cases, less than 50% of health professionals showed good attitude towards reporting ADR [15]. Also, the lack of knowledge on how to report an ADR and lack of access to reporting forms are major factors contributing to poor reporting rate [16].

Though health professionals have enough knowledge and are aware of the need to report ADR, smaller proportions spontaneously report ADR [17]. Health system factors that influence spontaneous reporting of ADR include health professionals knowledge, attitude and beliefs [18]. Socio-demographic characteristics of the health professional such as their age, gender, qualification, years of practice, number of patients seen in a day, and marital status also influence spontaneous reporting of ADR [19]. Elsewhere, female health professionals were more likely to report ADR than their male counterparts [19, 20]. The health professional’s age tends to affect reporting rate, which has been consistent across studies from different geographic locations with older and experienced professionals more likely to report an event [19, 21, 22].

The perennial issue of underreporting ADR in the pharmacovigilance system in Ghana and the consequential increase in mortality, hospitalization, congenital anomaly and economic burden as well as paucity of data influenced our decision to conduct this study in an attempt to identify context-specific factors influencing the rate of spontaneous ADR reporting among health professionals. It is anticipated that information obtained from this study would be useful to stakeholders in the formulation of policies and strategies to increase spontaneous reporting of ADR in the country. In Ghana, the Food and Drugs Authority (FDA) is responsible for drug safety issues under the Public Health Act 851. Thus, the Ghana FDA keeps an ADR data repository. The trend of spontaneous reporting has progressively increased since the country joined the International Medicine Safety Monitoring System in 2001 as the 65th Member State and the 1st in sub-Saharan Africa [23]. The Pharmacovigilance system of reporting in Ghana is such that, at the various health facilities there are mandated Institutional Contact Persons (ICP) who should serve as the link between the Ghana FDA and the health professionals with regards to ADR reporting [24]. Where a patient experiences an ADR, an ADR reporting form (known as the “blue form”), is filled and submitted through the ICP or any health professional at the facility to the regional offices of the Ghana FDA [24]. At the regional offices, there are designated regional Pharmacovigilance officers who receive the report forms, assess the validity of the report and present the report to the national FDA Central Database [23]. Health professionals from a health facility can forward an ADR report directly through the online reporting page of the FDA’s website to an International Conference of Harmonization E2B compliant Central Database of the Ghana FDA [24]. The reports received at the headquarters of Ghana FDA are forwarded as an E2B (xml) file [23].

While Health professionals report adverse events voluntarily, Pharmaceutical Manufacturing Companies are mandated to report ADR of their pharmaceutical products to the Ghana FDA. The companies do this through the Qualified Person for Pharmacovigilance (QPPV), a trained individual responsible for reporting all pharmacovigilance for all medicinal products by pharmaceutical companies holding marketing authorizations.

## Methods

### Study design

This study employed an analytical cross-sectional design using a well-structured, pretested, close-response questionnaire to collect data from randomly selected health professionals involved in ADRs reporting in the Kpone-Katamanso District in Ghana.

### Study population and location

The study population was health professionals in the Kpone-Katamanso District who were directly involved in the diagnosing, dispensing and administering drugs directly to the patients and as such are in the position to report any drug-related reactions of the patient. They included: pharmacists and pharmacy Technicians, physicians and physician assistants, nurses, midwives, and disease control officers working in health facilities in the Kpone-Katamanso District.

The Kpone-Katamanso District was created out of the Tema Metropolitan Assembly in 2012 through a legislative instrument. The district located on longitude 0°3′25″E and latitude 5°41′23″N along the coastal belt of Tema, about 38 km from Accra, the capital of Ghana, is made up of five sub-districts. The sub-districts are Kpone (the district capital), Zenu, Gbetsile, Oyibi and Appolonia. Kpone-Katamanso has a huge population of about 109,864 inhabitants with several health facilities that are equitably distributed [25]. As at 2010, the district had about 736 health professionals working in its health facilities [25].

### Inclusion/exclusion criteria

Health professionals with at least 1 year of experience at various health facilities, who interact directly with patients in relation to medicine use and are in a position to detect and report ADRs and also gave consent for participation were enrolled. Workers in the facilities who were solely employed for administrative duties and health workers on in-service or pre-qualification training were not included in this study.

### Sample size determination

The sample size was estimated using the Cochran formula [26], with an assumption that 16% of health professionals have ever reported adverse drug reaction. This assumption is based on a similar study conducted by Sabblah et al., which found that 16% of health professionals in the Volta region of Ghana reported an ADR [17], with 95% confidence interval (CI), 5% precision and 15% non-response rate. The final sample size was 245, representing the minimum number of participants required for this study. In all, a total of 363 professionals from 29 medical facilities as well as 34 community pharmacies in the District satisfied the inclusion criteria.

### Sampling technique

Health professionals were selected by multistage random sampling. A list of all health facilities in the various sub-districts was obtained from the Kpone Katamanso District Health Directorate. There were 29 medical facilities in the District comprising six health centers, nine clinics, one polyclinic, one maternal home, seven hospitals and four CHPS. Also, there were 34 community pharmacies in the District. At the first stage of sampling, the 29 medical facilities were numbered and 19 were randomly selected. Again, the 34 community pharmacies were numbered and 17 community pharmacies were randomly selected using an online random number generator with permission from the authorities of these facilities. A total of 363 participants met the inclusion criteria for this study. However, after simple random sampling, where the eligible health professionals were made to pick from an envelope containing 800 shuffled sheets with equal proportions of ‘Yes’ and ‘No″ written on them. This random sampling technique was adopted from Arora et al. [27]*.* The randomly selected participants were informed on the rationale, ethical issues, and purpose of the study. They were also assured of privacy and confidentiality. Participants were permitted to ask questions. Informed consent was obtained from all the randomly selected participants before they were enrolled into the study.

### Data collection and management

The data were collected using a closed-ended questionnaire developed after reviewing related studies and previous practice experience. The questionnaire captured the health professionals’ demographics, knowledge of health professionals on spontaneous reporting, the attitude of health professionals towards reporting, ADR reporting practice, and the factors that they perceived may influence reporting. Provision was also made for suggestions on possible ways to improve ADR reporting. The dependent variable, Spontaneous of ADR, was measured as a 4-item composite variable. These items were; having observed a patient with an adverse reaction, reported adverse drug reaction, stated reporting of ADR to Food and Drugs Authority (FDA), having received a feedback from the FDA and documented the observed ADR. A score of 1 was awarded to each correct item. A Health professional was said to have reported an ADR if the respondent had ever witnessed an ADR, reported to FDA, since they are responsible for pharmacovigilance in Ghana and had received a feedback from FDA as stipulated in the FDA’s Druglens Newsletter (10) or had documented the ADR as required by practice that health professionals should document clinical activities at all times. Hence, a score equal to or more than three means the participant had reported ADR before.

The proportion of health professionals who spontaneously reported an ADR was determined by dividing participants who had ever reported ADR by the number of participants assessed. To measure knowledge of participants, 6 items were used and every correct answer was coded as 1, otherwise 0 for a wrong answer. A score of 5 to 6 was considered as Excellent 4 to 3 was Adequate, and 2 to 0 was rated as Poor knowledge of a participant on adverse drug reporting. The variable, knowledge was measured as an ordinal one. To measure attitude of professionals as poor or good, 12 items were used. A coding of 1 was scored for ticking Yes and 0 for ticking No. Attitude was classified as poor, if the respondent` scored a value greater than or equal to 6 whilst it was classified as good if the respondent scored a value less than 6. The factors affecting ADR reporting among community pharmacists were assessed based on multiple responses to questions like Concern that the ADR report may be wrong, availability of reporting forms, fear of legal consequences, the workload on participants, etc. These factors were coded 1 for Yes and 0 for No. This provided nominal variables, which were used for the univariate analysis. Data collected were checked, cleaned, coded and entered into Stata version 15. The data were verified independently for errors and accuracy.

The researcher of this study encrypted the dataset with password protection and securely stored a copy of the dataset on a study designated pendrive kept in a safe. The paper questionnaires were shredded after the data were locked and securely stored on the pendrive.

### Data processing and analysis

Descriptive statistics were used to summarise the socio-demographic characteristics of the study participants. Categorical variables were summarised using frequencies and percentages. A simple logistic regression analysis was carried out to determine the association between the dependent variable (spontaneous reporting of ADR) and the independent variables (unadjusted odds ratio). Multiple logistic regression analysis was then used to assess the association between variables that showed to be statistically significant under the Chi-square test and simple logistic regression analysis. The estimates were expressed with their respective 95% Confidence Intervals (CIs) and *p*-values. A *p*-value < 0.05 was considered statistically significant.

### Ethical considerations

Ethical approval for the study was obtained from the Ghana Health Service Ethics Committee (Reference - GHS-ERC032/01/18). Informed consent was obtained from the participants of the study. The researchers ensured that all health professionals were adequately educated on the purpose of the study, risks and benefits of the conduct of the study and the health professionals were allowed to ask questions before their consent was taken. The health professionals were assured of The confidentiality and privacy during the conduct of the study. The study procedures were carried out in accordance with relevant guidelines and regulations.

## Results

### Demographic characteristics of participants

All 268 health professionals (Table 1) enrolled in the study responded to the questionnaire, giving a response rate of 100%. There were 119 (44.4%) males and 149 (55.6%) females. The representation of the different professionals involved in the study sample were: 35 Medical Officers (13.1%), 21 Pharmacists (7.8%), 53 Pharmacy Technician (19.8%), 93 Nurses (34.7%), 31 midwives (11.6%), 32 Physician Assistants (11.9%) and 3 Disease Control Officers (1.1%), and the average age was 34.1 (SD = 0.47). The oldest age recorded among the 268 participants was 60 years, and the youngest was 20 years. The least experienced had a one-year record of practice, and the most experienced had 36 years of practice; the average years of practice of respondents was 8.5 (SD = 0.62). From the data collected, 208 (77.6%) of the health professionals in the District had observed at least one case of ADR, whereas only 36 (17.3%) had ever reported an ADR to the Ghana FDA. Of the 268 participants, 46 (17.1%) had poor knowledge, 139 (51.9%) had average knowledge, and 83 (31.0%) had good knowledge of spontaneous reporting of ADR.

**Table 1:** Socio-demographic Characteristics of Health professionals from Kpone-Katamanso District, Ghana

Background Characteristics	Mean, (SD)	Number	Percentage (%)
**Gender**			
Male		119	44.4
Female		149	55.6
**Age (years)**			
Less than 30		76	28.36
30–39	34.13, (0.472)	139	51.87
40–49		39	14.55
50–59		8	2.99
Greater than 59		6	2.24
**Profession**			
Medical Officer		35	13.06
Pharmacist		21	7.84
Nurse		93	34.7
Pharm Tech		53	19.78
Midwife		31	11.57
Physician Assistant		32	11.94
Disease Control Officer		3	1.12
**Institution**			
Government		88	32.84
Private Hospital		167	62.31
Community Pharmacy		13	4.85
**Religion**			
Christian		234	87.97
Muslim		25	9.13
Other		8	3.01
**Years of Practice**			
Less than 10		194	72.39
10–19		50	18.66
20–29		18	6.72
Greater or equal to 30		6	2.24
**Marital Status**			
Married		165	61.57
Single		87	32.46
Divorced		16	5.97
**Witnessed a case of ADR**			
Yes		208	77.61
No		60	22.39
**Spontaneous Reporting**			
Yes		36	13.43
No		232	86.57

### Factors influencing spontaneous reporting of ADR

Table 2 presents the results of the simple logistic regression models, showing the factors associated with spontaneous reporting of ADR. Age was independently significantly associated with spontaneous reporting of ADR. It was realized that health professionals who were more than 50 years old were 8.8 times more likely to report an adverse event compared to professionals who were less than 30 years old (Crude Odds Ratio [COR] = 8.75; CI = 2.27–33.67). The field of Health Profession was also found to be independently associated with the spontaneous reporting of ADR. Nurses were found to be 84% less likely to report an adverse event compared to medical doctors (COR = 0.16; CI = 0.05–0.53). Pharmacy technicians were also 9.1 times less likely to report an adverse event compared to Medical Doctors (COR = 0.11; CI = 0.02–0.56). The level of knowledge of health professionals was significantly associated with Spontaneous Reporting of ADR. Health professionals with Excellent Knowledge of Spontaneous ADR reporting are 14.2 times more likely to report an ADR (COR = 14.17; CI = 1.83–109.67). Under professional attitudes, it was realized that Health professionals who are Uncertain about the cause of ADR were 68% less likely to report an adverse event compared to professionals who are certain of an ADR (COR = 0.32; CI = 0.15–0.70). Health professionals who had inadequate time at work were 4.3 times less likely to report an ADR compared to health professionals who had adequate time (COR = 0.23; CI = 0.11–0.48). Health professionals who were afraid of Legal consequences after reporting an ADR were found to be 84% less likely to report an ADR. Compared to health professionals who were not afraid of any legal consequences (COR = 0.16; CI = 0.06–0.37). Lastly, under the health professional’s attitude, it was found that health professionals who are diffident of the reporting procedure were 71% less likely to report an ADR compared to health professionals who are not diffident about the ADR reporting procedure (COR = 0.29; CI = 0.14–0.62). Under the stakeholder’s factors, health professionals who were not aware of the availability of reporting form were 79% less likely to report an ADR compared to health professionals who were aware of the availability of the reporting form (COR = 0.21; CI = 0.09–0.48). Health professionals who receive Prompt feedback from the FDA are 2.4 more likely to report an ADR compared to health professionals who do not receive (COR = 2.38; CI = 1.38–4.98), and health professionals who received Pharmacovigilance training were 19 times more likely to report an ADR compared to health professionals who did not receive any Pharmacovigilance training (COR = 19.04; CI = 7.07–51.27).

**Table 2:** Simple logistic regression of spontaneous adverse event reporting and related factors

VARIABLES	COR, 95% CI
Socio-Demographic	
Sex	
Male	1
Female	0.77, (0.38–1.56)
Age (years)	
Less than 30	1
30–39	1.63, (0.61–4.31)
40–49	2.55, (0.79–8.21)
Greater than 50	**8.75, (2.27–33.67)**^**a**^
Profession	
Medical Officer	1
Pharmacist	2.17, (0.69–6.84)
Nurse	**0.16, (0.05–0.53)**^**a**^
Pharm Tech	**0.11, (0.02–0.56)**^**a**^
Midwife	0.69, (0.22–2.23)
Physician Assistant	0.30, (0.07–1.22)
D. Control Officer	5.78, (0.47–71.62)
Institution	
Government	1
Private Hospital	1.23, (0.58–2.64)
Community Pharmacy	0.54, (0.06 - 4.53)
Religion	
Christian	N/A
Muslim	0
Other	0
Knowledge	
Poor	1
Average	6.16, (0.80–47.8)
Good	**14.17, (1.83–109.67)**^**a**^
VARIABLES	COR, 95% CI
Professional Attitudes	
Uncertainty about cause of ADR	
No	1
Yes	**0.32, (0.15–0.70)**^**a**^
Lack of Adequate time	
No	1
Yes	**0.23, (0.11–0.48)**^**a**^
Diffident about ADR occurrence	
No	1
Yes	0.71, (0.34–1.49)
Fear of legal repercussions	
No	1
Yes	**0.16, (0.06–0.37)**^**a**^
Extraneous work	
No	1
Yes	1.20, (0.55–2.54)
Ambitious to publish a case report	
No	1
Yes	0.62, (0.27–1.43)
Mistrust in PV system	
No	1
Yes	0.56, (0.27–1.13)
Negligence	
No	1
Yes	1.0, (0.49–2.03)
Diffident about Procedure	
No	1
Yes	**0.29, (0.14–0.62)**^**a**^
Indifferent about reporting	
No	1
Yes	1.87 (0.87–4.0)
Lack of incentives	
No	1
Yes	0.94, (0.46–1.94)
Form of ADR reactions	
Seriousness of reaction	
No	
Yes	N/A
Unusual reaction	
No	1
Yes	1.63 (0.78–3.40)
Unknown Reaction to product	
No	1
Yes	0.95 (0.47–1.92)
Stakeholder Factor	
Unavailability of reporting forms	
No	1
Yes	**0.21 (0.09–0.48)**^**a**^
Prompt Feedback	
No	1
Yes	**2.38 (1.13–4.98)**^**a**^
PV in curriculum	
No	1
Yes	1.10 (0.53–2.26)
Post-professional PV Training	
No	1
Yes	**19.04 (7.07–51.27)**^**a**^

^a^Statistically significant

*COR* Crude Odds Ratio

*CI* Confidence interval

Table 3 presents the results of the multivariate logistic regression models, showing the factors associated with spontaneous reporting of ADR. After adjustment on potential confounding variables (Knowledge, Feedback from FDA, Uncertainty about cause of ADR, Severity of ADR), Age: 40–49 (AOR = 93.10, *p*-value < 0.01), Reporting by Nurses (AOR = 0.01, p-value < 0.01), the spontaneous reporting of ADR by Disease control officers (AOR = 12.38, p-value = 0.02); Professional attitudes such as Uncertainty about ADR report (AOR = 2.29, p-value < 0.01), No time for reporting (AOR = 0.3, p-value = 0.03), Fear of legal consequences after reporting (AOR = 0.15, p-value < 0.01) and Post qualification training (AOR = 18.78, p-value < 0.01); were statistically significant.

**Table 3:** Multivariate logistic regression of spontaneous adverse event reporting and related factors

VARIABLE	Adj. Odd ratio	95% CI	*P* value
Age (years)			
Less than 30 (Ref)	1		
30–39	**10.13**	**1.056–97.217**	**0.045**
40–49	**93.10**	**4.577–1893.7**	**0.003**^**a**^
Greater than 50	2.21	0.0925–52.947	0.624
Profession			
Medical Officer (Ref)	1		
Pharmacist	1.93	0.086–43.16	0.67
Nurse	**0.01**	**0.001–0.185**	**< 0.01**^**a**^
Pharm Tech	0.18	0.008–4.170	0.29
Midwife	3.31	0.161–67.852	0.43
Physician Assistant	.20	0.0073–5.152	0.32
Disease Control Officer	**12.382**	**1.8039–84.984**	**0.02**^**a**^
Knowledge of Professionals			
Poor (Ref)	1		
Average	7.967	0.335–189.246	0.2
Good	8.311	0.333–207.123	0.2
Uncertainty about cause of ADR			
No (Ref)	1		
Yes	0.34	0.11–1.06	0.06
Time constraint to report ADR			
No (Ref)	1		
Yes	**0.30**	**0.10–0.91**	**0.03**^**a**^
Fear of legal repercussions			
No (Ref)	1		
Yes	**0.15**	**0.41–0.51**	**< 0.01**^**a**^
Diffident about reporting procedure			
No (Ref)	1		
Yes	0.77	0.26–2.33	0.65
Unavailability of Reporting form			
No (Ref)	1		
Yes	**0.28**	**0.09–0.88**	**0.03**^**a**^
Feedback from FDA			
No (Ref)	1		
Yes	2.12	0.70–6.37	0.181
Pharmacovigilance training			
No (Ref)	1		
Yes	**18.78**	**5.46–64.59**	**< 0.01**^**a**^

^a^Statistically Significant

*AOR*Adjusted Odds Ratio

**Fig. 2:**
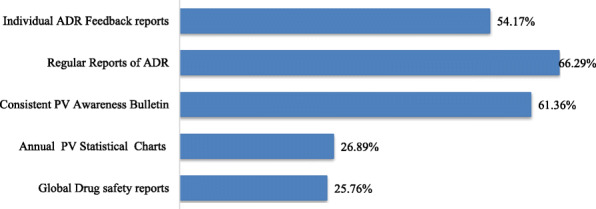
Expected Forms of Feedback from Pharmacovigilance System

Figure 2 represents suggested forms of feedback that could improve spontaneous reporting of adverse drug reaction by the Health professionals of Kpone-Katamanso District. From the figure, 66.29% of respondents suggested that the Food and Drugs Authority produce regular reports on recent Adverse Drug Reactions. In addition, 61.36% of the 268 respondents believed that Regular Pharmacovigilance Awareness reports and programs could help improve the spontaneous reporting rate of Adverse Drug Reaction. Some respondents (54.17%) believed that individual feedback by the Ghana FDA to ADR reporting could go a long way to enhance reporting of ADR. 26.89% suggested that annual statistic charts of cases of Adverse Drug Reactions would improve the feedback from the Ghana FDA, while 25.76% of respondents would prefer a Global Drug Safety reports as feedback from the Food and Drugs Authority.

**Fig. 3:**
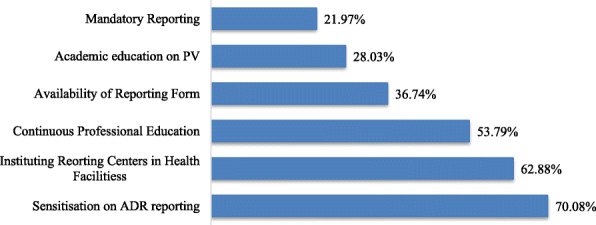
Recommended Ways to improve ADR Among Health Professionals

On recommended ways to improve Spontaneous Reporting, most of the respondents (70.08%) recommended that there be awareness creation and sensitization through media. 62.88% of the respondents also believe that establishing Institutional Reporting Centres would improve ADR reporting rate. 53.78% of respondents recommended Continuous Professional Education and Training. About a third of professionals (36.74%) believe that the availability of reporting forms is integral for improving spontaneous reporting of adverse drug reaction. 28.03% of the respondents recommended that pharmacovigilance studies be incorporated into their educational curriculum (Fig. 3).

## Discussion

The contribution of healthcare providers in reporting ADR is paramount to the sustenance of every pharmacovigilance system. This study provides germane insight regarding knowledge, practice and impetus of health professionals towards ADR reporting as well as its associated factors. This study revealed that only 36 (17.3%) out of 208 health professionals who had witnessed an ADR in the Kpone-Katamanso District had ever spontaneously reported it to the Ghana FDA.

Assessing the factors that may influence the abysmal spontaneous reporting of ADR amongst health professionals in the District divulged that the age of health professionals is significantly associated with reporting ADR. Health professionals aged between 30 and 39 were 10.13 times more likely to report an ADR spontaneously than professionals younger than 30 years. In addition, professionals aged between 40 and 49 years are 93.10 times more likely to report an adverse drug reaction than professionals younger than 30 years. It may be implied that as health professionals get older, they tend to gain more experienced and are often conversant with reporting procedures.

A factor that could be accounting for the low reporting of ADR, as deduced from this study, is the availability of ADR reporting forms. Health professionals who do not have access to reporting forms are 3.6 times less likely to report an ADR compared to their counterparts with reporting forms available at their health facilities. This finding is consistent with that of Eweuko, which indicated that lack of reporting forms at the various health facilities hampered the rate of spontaneous reporting of adverse drug reactions among health professionals [28].

Time constraint was identified as a factor hindering Spontaneous reporting of ADR [12, 13]. The Annual fact and figures Statistical Report of Ghana reveals a low health professional to patient ratio implying an increase in job demand on health professionals [29]. It was observed that health professionals who complained of time constraint were 6.7 times less likely to report an ADR than those who did not complain of time constraint. Professionals should be educated on the relevance of spontaneous ADR reporting in ensuring safe medicines for the management of conditions. Regardless of the workload on professionals, they should be educated to view spontaneous reporting with equal importance in ensuring proper patient care. This is because a single report of an ADR may save numerous lives.

A significant proportion of Health Professionals attributed the hesitation in reporting ADR to fear of legal repercussions similar findings were reported in other studies [30]. Perception of professional error seems to be a major cause of this fear. Health Professionals are scared that they may be prosecuted if ADR experienced are due to administering the wrong drug or wrong diagnosis. After reporting an ADR, assurance of legal protection would go a long way to increase reporting amongst Healthcare providers provided due procedures were adhered to. In addition, stakeholders should assure reporters of confidentiality and anonymity in order to boost the ADR reporting rate.

The study found that training on pharmacovigilance improves ADR reporting as health professionals who received some form of training on pharmacovigilance were 5.5 times more likely to report an adverse reaction than those with no training on pharmacovigilance. Pharmacovigilance training was significantly associated with spontaneous reporting of ADR. These findings are consistent with that of Sabblah et al. [17], who also concluded that professionals’ post-professional training positively impacted spontaneous reporting rates. It has been realized that healthcare professionals’ pharmacovigilance training and education is one of the strategies proposed to increase ADR reporting rates [17].

Low rates of feedback from authorities on submitted ADR forms could account for health professionals’ lackadaisical attitude to reporting of ADR. A study by Toklu et al. [31] disclosed that nearly 80% of pharmacists in Cyprus strongly approve that feedback to reports received from the National Pharmacovigilance Centre will go a long way to motivate them to report adverse drug reactions. It was imperative to find out from the respondent’s opinions on which forms of feedback would motivate them to report an adverse drug reaction. More than half of respondents from this study suggested that the Ghana FDA produce a regular newsletter on recent ADR. Yearly, the Ghana FDA releases a newsletter called the Druglens, which seeks to inform health professionals on strides made in the area of spontaneous reporting. These newsletters are disseminated to the various health facilities and made accessible to all health professionals. However, a survey by the FDA revealed that 38.3% of Health professionals were aware of at least one of the letters distributed to all professionals in 2013 [24]. This finding indicates that most professionals are not aware of any news alert on ADR and other relevant information to improve spontaneous reporting of ADR.

On recommended ways to improve Spontaneous ADR Reporting, studies have shown a paucity of empirical data on pharmacovigilance awareness in Africa [32]. Therefore, it is not surprising that most of the respondent (70.1%) recommended that there be sensitization on ADR reporting through media on the need for health professionals to report ADR. This recommendation is very laudable because sensitization through the media encourages reporting among consumers. Sometimes, especially at the community pharmacy, professionals depend on reports received from consumers to report an ADR. Nwokike & Eghan recommended media publicity following a review of Ghana’s pharmacovigilance system [33]. If these reports are not received or are lacking in vital information for assessments, it negatively affects the professional’s ability to report adverse drug reaction.

Over 50% of the respondents also believe that establishing ADR Reporting Centers at the various health facilities would improve the ADR reporting rate (Fig. 3). The Ghana FDA has introduced Institutional Contact Person (ICP) on pharmacovigilance who may be health professionals serving as a liaison between the Ghana FDA and the health professionals at the various institutions. This is a measure to improve spontaneous reporting of ADR among health professionals. Health professionals are now able to report ADR directly to the ICP. Nonetheless, there should be Institutional Reporting Centers (IRC), which the ICP could head. This would increase professionals’ awareness of the need to report ADR. It would also reduce the fear of legal repercussions after reporting since professionals who report are guaranteed anonymity and confidentially from the regulators (FDA).

A proportion of 53.78% of respondents recommended Continuous Professional Education and Training to be a sure way of improving spontaneous reporting of ADR. Continuous Professional Development (CPD) can be very beneficial for one’s career. Undertaking a CPD programme is a mandatory requirement for some professionals. It helps by advancing the knowledge and skills; professionals need to be confident that they are working at the top end of their profession. Pharmacovigilance training could be held during continuous professional development programs to train professionals on spontaneous reporting. It also offers an appropriate platform to assure professionals that there is no legal liability for reporting an adverse reaction. Professionals would also get adequate opportunity to ask questions pertaining to the spontaneous reporting of ADR.

This study is subject to recall bias as some health professionals were unable to adequately remember whether they reported or documented the adverse drug reactions. The information obtained is subjective since some respondents may give false information to look good or be perceived as professionals.

## Conclusion

Though most professionals demonstrated average knowledge on spontaneous reporting of ADR, the proportion of professionals who report ADR was low. The knowledge of professionals did not affect their attitude towards spontaneous reporting of adverse drug reaction. A policy should be implemented to make spontaneous reporting of adverse drug reaction mandatory for health professions. Just as it is stipulated in Section 125 of the Public Health Act, 2012, Act 851, that manufacturers should mandatorily report adverse drug reaction [34].

Given the public health impact of adverse drug reaction, Regulators are called upon to intensify awareness through media sensitization and engagement of all relevant stakeholders on the need for the entire population to report adverse drug reactions they experience.

## Data Availability

The dataset used and/or analyzed during the current study are available from the corresponding author on reasonable request.

## Abbreviations

ADR: Adverse Drug Reaction
CHPS: Community–based Health Planning Services
CPD: Continuous Professional Development
FDA: Food and Drugs Authority
GHS: Ghana Health Service
MOH: Ministry of Health
IMU: International Monitoring Unit
ICP: Institutional Contact Person
IRC: Institutional Reporting Center
GHS: Ghana Health Service
QPPV: Qualified Person for Pharmacovigilance
WHO: World Health Organization
